# Effect of the Trigeminal Nerve Stimulation on Auditory Event-Related Potentials

**DOI:** 10.1093/texcom/tgab012

**Published:** 2021-02-19

**Authors:** Maria Paola Tramonti Fantozzi, Fiorenzo Artoni, Marco Di Galante, Lucia Briscese, Vincenzo De Cicco, Luca Bruschini, Paola d’Ascanio, Diego Manzoni, Ugo Faraguna, Maria Chiara Carboncini

**Affiliations:** Department of Translational Research and of New Surgical and Medical Technologies, University of Pisa, Pisa 56123, Italy; Bertarelli Foundation Chair in Translational Neuroengineering, Center for Neuroprosthetics, Institute of Bioengineering, School of Engineering, École Polytechnique Fédérale de Lausanne, Genève 1202, Switzerland; SleepActa s.r.l., Pontedera 56025, Italy; Department of Translational Research and of New Surgical and Medical Technologies, University of Pisa, Pisa 56123, Italy; Department of Translational Research and of New Surgical and Medical Technologies, University of Pisa, Pisa 56123, Italy; Department of Surgical, Medical, Molecular Pathology and Critical Care Medicine, University of Pisa, Pisa 56123, Italy; Department of Translational Research and of New Surgical and Medical Technologies, University of Pisa, Pisa 56123, Italy; Department of Translational Research and of New Surgical and Medical Technologies, University of Pisa, Pisa 56123, Italy; Department of Translational Research and of New Surgical and Medical Technologies, University of Pisa, Pisa 56123, Italy; Department of Developmental Neuroscience, IRCCS Fondazione Stella Maris, Pisa 56128, Italy; Department of Translational Research and of New Surgical and Medical Technologies, University of Pisa, Pisa 56123, Italy

**Keywords:** acoustic oddball, locus coeruleus activity, pupil size, P300, trigeminal nerve stimulation

## Abstract

Trigeminal sensorimotor activity stimulates arousal and cognitive performance, likely through activation of the locus coeruleus (LC). In this study we investigated, in normal subjects, the effects of bilateral trigeminal nerve stimulation (TNS) on the LC-dependent P300 wave, elicited by an acoustic oddball paradigm. Pupil size, a proxy of LC activity, and electroencephalographic power changes were also investigated. Before TNS/sham-TNS, pupil size did not correlate with P300 amplitude across subjects. After TNS but not sham-TNS, a positive correlation emerged between P300 amplitude and pupil size within frontal and median cortical regions. TNS also reduced P300 amplitude in several cortical areas. In both groups, before and after TNS/sham-TNS, subjects correctly indicated all the target stimuli. We propose that TNS activates LC, increasing the cortical norepinephrine release and the dependence of the P300 upon basal LC activity. Enhancing the signal-to-noise ratio of cortical neurons, norepinephrine may improve the sensory processing, allowing the subject to reach the best discriminative performance with a lower level of neural activation (i.e., a lower P300 amplitude). The study suggests that TNS could be used for improving cognitive performance in patients affected by cognitive disorders or arousal dysfunctions.

## Introduction

Trigeminal afferents play a particularly important role in the control of arousal/alertness and attention through their connections with structures belonging to the Ascending Reticular Activating System (ARAS) (Roger et al. 1956). Trigeminal primary and/or secondary neurons project to the pontomedullary reticular formation, to the cholinergic pedunculopontine and the laterodorsal tegmental nuclei, to the histaminergic tuberomammillary, and to the noradrenergic locus coeruleus (LC) neurons (De Cicco et al. 2018). In particular, trigeminal mesencephalic nucleus (Me5), including the cell bodies of spindle afferents in masticatory muscles and of mechanoreceptors in the periodontal ligament (Yoshida et al. 2017), projects to LC (Luo et al. 1991) that affects vigilance via its mainly ipsilateral projections to cortical regions (Waterhouse et al. 1983) and influences cognitive processes (Usher et al. 1999).

Accordingly, in humans, chewing quickens cognitive processing (Hirano et al. 2013) and improves arousal (Allen and Smith 2012; Johnson et al. 2012) and attention (Tucha et al. 2004), leading to an increment of blood perfusion in several cortical and subcortical structures (Hirano et al. 2013). Moreover, chewing can prevent degenerative process in older animals (Chen et al. 2015) and, possibly, in humans, where it is related to preserved cognitive functions (Moriya et al. 2011), thus prompting its use in the field of neurorehabilitation for contrasting the cognitive decline associated to age-related neurodegenerative processes (Wilson et al. 2010).

In order to exploit the potential protective effects of mastication on the brain, the subjects have to be motivated to perform this activity, a condition that can be absent in individuals affected by cognitive impairments or by disorders of consciousness. This problem requires a noninvasive method for eliciting trigeminal activation, mimicking—to some extent—the sensorimotor signals elicited during chewing, by-passing the subject’s cooperation.

In this respect, the trigeminal nerve stimulation (TNS) applied to the mandibular branch—currently used in the gnathology practice for inducing masticatory muscle relaxation and achieving a correct occlusal contact between the dental arches (Nnoaham and Kumbang 2008; De Cicco 2012)—activates the large proprioceptive fibers that fire rhythmically during mastication (Goodwin and Luschei 1975).

An established quantitative index of cognitive processing is represented by the P300 wave, an event-related potential (ERP), consisting in a parietal–central positivity occurring together with the conscious detection of stimuli carrying important information about the performed task (Huang et al. 2015). This ERP has been largely studied and can be elicited in populations of patients with minimal or absent levels of collaboration (Polich and Corey-Bloom 2005; Parra et al. 2012; Pedroso et al. 2012; Wang et al. 2017; Zhang et al. 2017). Although several factors may affect the P300 amplitude, in memory recall tasks of written words, its size was related to task performance (Polich 2007). More specifically, the recall of a word dispersed among others of slightly different font was easier for those words eliciting P300 with a larger amplitude. This suggests an association between P300 amplitude and subsequent recognition performance.

In particular, the P300 wave can be elicited during an acoustic oddball paradigm, when a subject detects infrequent target stimuli randomly presented in a train of standard stimuli; it shows a latency of approximately 250–400 ms from stimulus onset (Huang et al. 2015). In this condition, the P300 amplitude reflects the amount of brain activity related to updating the mental stimulus representation in response to the target stimuli (Polich 2007; van Dinteren et al. 2014). In parallel, the P300 latency is related to stimuli classification speed, which reflects the time required for the sensory processing leading to target recognition (Polich 2007; van Dinteren et al. 2014). Consistently, the P300 and the associated detection performance are lower in patients affected by Alzheimer’s disease and schizophrenia (Jeon and Polich 2003; Hedges et al. 2014).

The P300 can be recorded over extended frontal–central–parietal regions and its rather stereotyped latency suggests that it could be attributed to a diffuse system synchronizing the activity of several cortical regions (Nieuwenhuis et al. 2005). There is indeed evidence that at least some components of this wave are related to the phasic LC activity elicited by the stimulus, which induces a widespread noradrenaline release at cortical level. In particular, the decrease of norepinephrine release by clonidine in humans leads to a reduced P300 amplitude and target recognition in an acoustic oddball paradigm (Nieuwenhuis et al. 2005). In this respect, it has to be noted that the activity level of LC neurons can be inferred by pupil size recordings (Rajkowski et al. 1994; Murphy et al. 2014). For this reason, the relation between pupil size and P300 amplitude was investigated in several studies: despite baseline pupil size was related to the P300 amplitude by an inverted-U relation (Murphy et al. 2011; Hong et al. 2014), the pupil dilation during the task did not correlate with the P300 amplitude (Kamp and Donchin 2015).

In conclusion, the P300 is an indicator of cognitive processing and depends upon the noradrenergic system, whose activity is strongly influenced by sensorimotor trigeminal activity. For these reasons, in order to clarify the suitability of TNS in boosting cognitive performance through LC activation, we studied, in healthy volunteers, its effects on the P300 wave elicited during the acoustic oddball task as well as on the electroencephalographic (EEG) activity recorded at rest. Moreover, since LC activity is closely related to pupil size, the effects of TNS on pupil diameter were also evaluated.

## Materials and Methods

This study was approved by the Ethical Committee of the University of Pisa (approval no. 12/2019). According to the Declaration of Helsinki, each subject signed an informed consent.

### Subjects

Experiments were carried out in 13 voluntary healthy subjects (8 females) aged between 24 and 31 years (mean ± standard deviation [SD], age: 27.4 ± 2.4), not affected by pain in the masticatory/neck muscles and by neurological, psychiatric, metabolic, or endocrine diseases.

### Experimental Procedure

**Figure 1:**
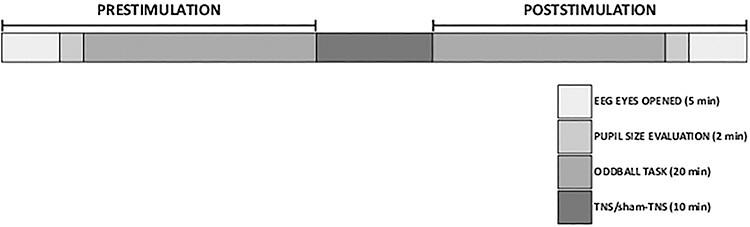
Time course of the experimental protocol. The line represents the whole experimental protocol and each box corresponds to a specific step. For each step the duration expressed in minutes is reported.

Participants were seated in a comfortable armchair located in the laboratory. One couple of recording electrodes for surface electromyography (EMG) recordings was applied over the masseter belly on both sides, and the subjects were prepared for the EEG recording. Each session started with a 5-min eyes-open recording. In the eyes-open condition, participants were instructed to fixate a small cross presented on the wall in front of them. Then, the pupil size was evaluated. Since the occlusal condition strongly affects the pupil diameter (De Cicco et al. 2014, 2016), recordings were taken both with the arches slightly apart and in contact. At this point, a 20-min auditory oddball task was performed. For this purpose, subjects wore acoustic headphones through which a random sequence of frequent standard tones (*n* = 400) and rare (target) oddball tones (*n* = 100) were simultaneously delivered to both ears. Rare and frequent stimuli were pure tones lasting 50 ms at 2000 and 1000 Hz frequencies, respectively. Participants were instructed to mentally count the rare tones and, at the conclusion of the oddball, to report their number. Subsequently, TNS was performed in 7 subjects (4 females, age: 26.9 ± 2.0) and sham-TNS in another sample of 6 subjects (4 females, age: 28.0 ± 2.9). Soon after the end of TNS/sham-TNS the oddball task was repeated, followed by pupil size and eye-open EEG recordings (Fig. 1).

### Trigeminal Nerve Stimulation

TNS consisted of 10-min bilateral transcutaneous stimulation of the trigeminal motor branches through couples of electrodes applied at the level of incisura sigmoidea, leading to small and symmetrical mandibular movements (cathodal/anodal current pulses, 0.54 ms, 21–25 mA, 0.618 Hz). The amplitude and frequency of stimulation used in the present experiments were well below the pain threshold. The intensity of the right and left current was adjusted to obtain a symmetrical EMG response (amplitude) of the masseter muscles on both sides.

### Sham-TNS

In the sham-TNS condition, electrodes were applied at the level of the incisura sigmoidea of both sides, but no stimulation was given for 10 min.

### Pupil Size Evaluation

Pupil diameter was recorded from both eyes in standard condition of artificial lighting by using a corneal topographer–pupillographer (MOD i02, with chin support, CSO), under standard illumination (halogen lamp, white light, ensuring a constant luminance level) while continuously recording pupil size via a camera sensor CCD1/3”, with a 56-mm working distance and at a sampling rate of 120 frames per second (fps). Measurements were triggered with 33 ms of delay with respect to the time the constant level of lightning provided by the device (40 lux) was switched off and before the beginning of pupil dilatation (~300 ms from light off; Bitsios et al. 1996).

Only the initial frame from light-off was analyzed. Measurements were taken in the following order:

1. with the dental arches 1–2-mm apart, without chin support;
2. with the dental arches in contact, the chin being supported.

Only measurements taken in position 2 were utilized for the analysis (see Results section).

Diameter values were stored for further analysis and displayed online on the computer screen.

### Electrophysiological Recordings and EEG Data Analysis

EEG data were acquired at the sampling rate of 1024 Hz from 20 gold-plated cup electrodes (Fp1, Fpz, Fp2, F7, F3, Fz, F4, F8, T3, C3, C4, T4, T5, P3, P4, T6, O1, Pz, O2, and Cz), affixed with EC2 paste (EC2® Genuine Grass Electrode Cream, Grass Instruments), taped on the scalp and positioned according to the 10–20 International Electrodes Placement System. The reference was placed on a common neutral auricular derivation. Data were analyzed via custom routines based on EEGLAB toolbox (Delorme and Makeig 2004) and Matlab R2017b. Offline, the whole EEG signals were digitally band-pass filtered between 0.5–30 Hz and re-referenced to T5. The EEG data recorded during the oddball tasks and at rest were processed for ERPs and power spectrum density (PSD) analysis, respectively.

#### Event Related Potentials

The EEG data were segmented into epochs time-locked to the onset of the stimuli, with pre- and poststimulus time windows of 0.5 and 1 s, respectively. The average baseline, prestimulus value was subtracted from each trial. Epochs with large prominent artifacts were removed by visual inspection followed by Independent Component Analysis (ICA) (Hyvärinen and Oja 2000), for stereotyped artifacts elicited by ocular movements and muscle activation. EEG signals related to standard and target stimuli were separately averaged before and after TNS/sham-TNS.

**Figure 2:**
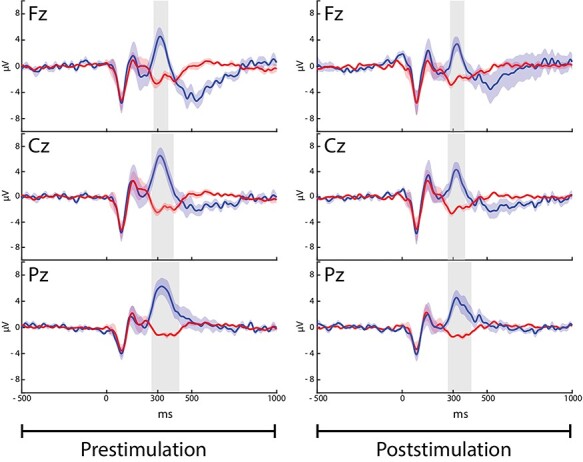
Grand average (*n* = 7) of ERPs waveforms measured in Fz, Cz, and Pz electrodes within the TNS group. Blue and red traces represent responses to the rare and frequent standard stimuli, respectively. The color-coded shadowed regions around the traces correspond to standard error (SE) values. Significant differences between rare and frequent responses over the interval 250–400 ms identify the P300 event and are here highlighted by the gray areas (Table 1).

For ERPs analysis, the amplitude and latency of N100, P200, and P300 were extracted from 19 electrodes for each subject. Figure 2 shows the grand average obtained for the whole population of TNS group at 3 different derivations (Fz, Cz, Pz), before and after stimulation.

The amplitudes of the N100, P200, and P300 peaks were determined using an automatic local peak detection relative to different time windows as follows. N100 and P200 reflect the neural processes that are sensitive to sound stimulus features (intensity, frequency) (Remijn et al. 2014). As a routine procedure, N100, P200, and P300 were identified as the most negative (for N100) and positive (for P200 and P300) peaks in the 0–130, 150–250, and 250–400 ms time windows following stimulus onset, respectively. The latency of each ERP component was defined as the point in time of the peak with respect to the stimulus onset.

#### Power Spectrum Density

EEG resting data, relative to the 5-min periods in the eyes open condition, were processed by rejecting the same ICA components discarded during ERPs processing. The EEGLAB Matlab tool “spectopo” function was used for obtaining the PSD (in μv^2^) in all the EEG channels with a resolution of 1 Hz. Further analysis was clustered in standard delta (0.5–4 Hz), theta (4–8 Hz), alpha (8–13 Hz), and beta (13–30 Hz) bands.

#### Cognitive Performance

The cognitive performance was evaluated as the difference between the actual number of rare tones included in the oddball sequence and that reported by the subject.

### Statistical Analysis

The original database is publicly available online at the following address: osf.io/jsvnk.

#### Pupil Size Evaluation

The differences in average pupil size and anisocoria (individual absolute difference between right and left pupil size), evaluated with the dental arches in contact between the 2 groups (TNS/sham-TNS) or between pre- and poststimulation, were evaluated by independent and paired *t*-tests, respectively. Moreover, possible linear correlations between pupil size and P300 amplitude were analyzed using Pearson’s correlations.

#### Event-Related Potentials

Measurements were focused on N100, P200, and P300 peak amplitudes and latencies. P300 peak and latency were evaluated only for target stimuli. To investigate the changes in all the ERPs components between pre- and poststimulation, a paired sample *t*-test was used (Li et al. 2019) on the corresponding peak features. Moreover, a point-by-point comparison was performed between responses to rare and frequent stimuli in the whole poststimulus interval by *t*-test, in order to highlight the P300-related time interval, where the EEG signal relative to the rare stimulus response was significantly higher than that relative to the frequent stimulus (Table 1).

**Table 1:** P300 intervals

Stimulation type	Electrode	Rare vs. standard responses	
		P300 interval, prestimulation	P300 interval, poststimulation
TNS	Fz	275.4 ≤ ms ≤ 363.3	283.2 ≤ ms ≤ 365.2
	Cz	262.7 ≤ ms ≤ 393.6	268.6 ≤ ms ≤ 369.1
	Pz	263.7 ≤ ms ≤ 426.8	269.5 ≤ ms ≤ 407.2
sham-TNS	Fz	252 ≤ ms ≤ 347.7	260.7 ≤ ms ≤ 373
	Cz	252 ≤ ms ≤ 367.2	260.7 ≤ ms ≤ 421.9
	Pz	258.8 ≤ ms ≤ 417	260.7 ≤ ms ≤ 436.5

Note: P300 intervals detected in Fz, Cz, and Pz, pre- and post-TNS/sham-TNS conditions. In all the points of these intervals, a significant difference (*P* < 0.05) was found, point-by-point, between the response to rare and the frequent stimuli (Fig. 2).

#### PSD of EEG Open Eyes

Changes in the oscillatory EEG activity were investigated to evaluate the effects of TNS/sham-TNS on ongoing brain activity. In particular, for each electrode, pre- and post-TNS/sham-TNS spectral power was compared within the delta (0.5–4 Hz), theta (4–8 Hz), alpha (8–13 Hz), and beta (12–30 Hz) bands, with a resolution of 1 Hz bins, through a paired *t*-test.

**Figure 3:**
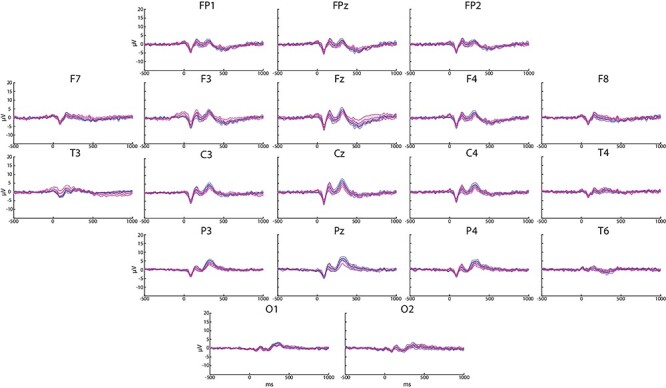
Grand average (*n* = 7) of ERP responses to rare stimuli for the TNS group recorded at all the electrodes. The blue and the magenta lines represent the pre- and the poststimulation waveforms, respectively. The shadowed regions around the traces correspond to SE values.

#### Cognitive Performance

The effects of TNS/sham-TNS on the number of errors made in the oddball paradigms were evaluated by paired *t*-test.

All statistical analyses were conducted with both Matlab—Statistical Toolbox and the Statistical Package for Social Sciences (SPSS, version 20). The level of statistical significance was set at *P* < 0.05. To control for possible false positives introduced by multiple comparisons, a bootstrap procedure was used. Subjects were randomly extracted from both TNS and sham-TNS groups for a number of times corresponding to the sample size of each experimental group (sham-TNS: *n* = 6; TNS: *n* = 7), so to generate new populations (either sham-TNS or TNS) where a given subject could be represented more than once. Within the populations so obtained, the amplitudes of P300 observed before and after TNS/sham-TNS were compared by paired *t*-test for each of the 19 electrodes. The random extraction from the 2 groups and the comparison of the P300 amplitudes was repeated 1000 times and the average *P* values and their 5% confidence intervals were evaluated in both groups for each electrode.

The same approach was implemented to compare the changes in EEG power induced by TNS/sham-TNS in the different frequency bands. Thus, data relative to each of the 1000 random extractions were used to perform 30 (frequency points) × 19 (electrodes) comparisons of the power values observed before and after TNS/sham-TNS.

## Results

### Event-Related Potentials

The individual and grand averages of scalp EEG signals recorded before and after TNS/sham-TNS revealed typical ERPs responses to the target stimuli consisting of N100 and P200 followed by P300 waveforms. The P300 was not found after frequent stimuli (Fig. 2) and showed a typical midline central–parietal distribution (Fig. 3) (Picton 1992). The extent of P300 intervals recorded at the different electrodes is shown in Table 1.

**Figure 4:**
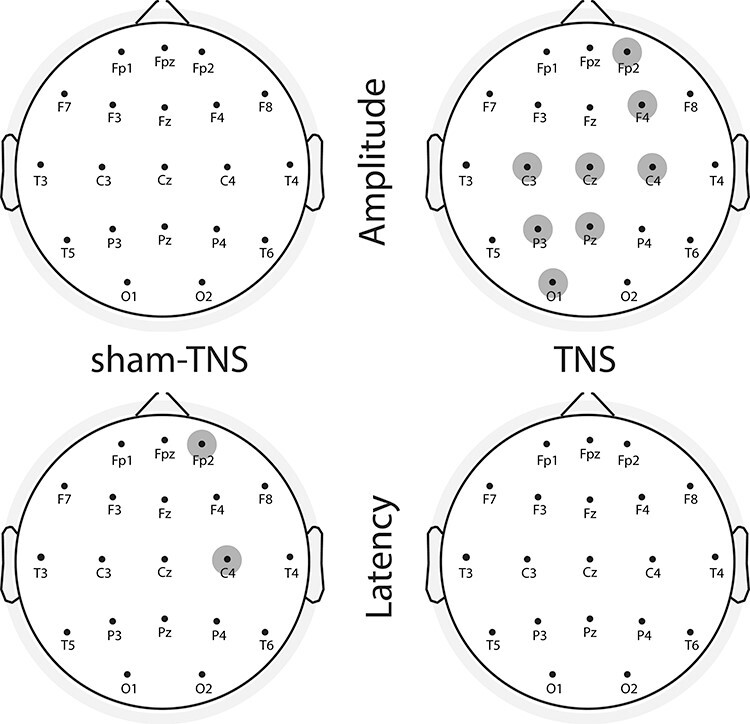
Results of the statistical analysis for the P300 component in sham-TNS/TNS groups. The electrodes showing significant differences (in amplitude or in latency) between pre- and post-stimulation are indicated by the highlighted electrodes.

*T*-test comparisons showed no differences in N100 and P200 amplitude and latency between pre- and post-TNS or sham-TNS, with the only exception of O1 and O2, where sham-TNS modified the amplitude of P200 (O1: from 2.1 ± 1.19 to 1.43 ± 0.97 μV, *P* = 0.022) and N100 (O2: from −1.22 ± 1.13 to −1.72 ± 1.35 μV, *P* = 0.010), respectively. As to the P300, this component showed a poststimulation amplitude decrease in the TNS group, statistically significant in 8 out of 19 electrodes (Fig. 4).

**Figure 5:**
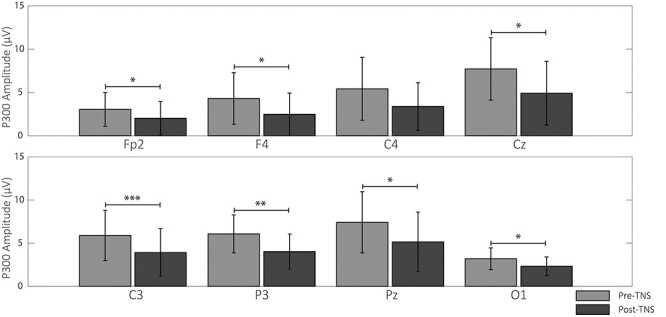
P300 amplitude before and after TNS. Mean ± SD of the P300 peak amplitude in pre- (light gray bars) and post- (heavy gray bars) TNS condition evaluated at the electrodes showing a significant pre–post difference, based on uncorrected *P* values. Asterisks indicate the significance level of the corresponding comparison after correction through bootstrap procedure (^*^P < 0.05^*^^*^; *P* < 0.01^*^^*^^*^; *P* < 0.005). See text for further explanations.

Following the bootstrap-based procedure the significance was maintained at all these electrodes, but C4 (*P* = 0.093) (Fig. 5).

**Figure 6:**
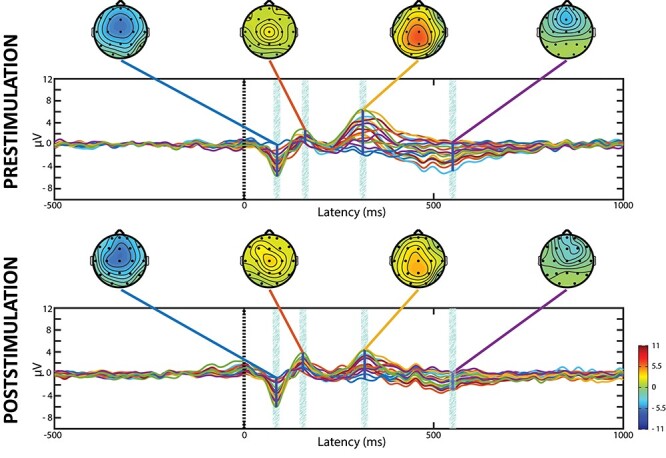
Grand average ERPs evaluated in the TNS group for each of the 19 channels investigated have been superimposed before and after the stimulus. The scalp maps show the topographic distributions of voltage value, color-coded and recorded at the times of N100, P200, and P300 peaks and at 550-ms poststimulus onset.

As for the P300 latency, this changed only at FP2 (from 310.22 ± 35.99 to 322.43 ± 31.54 ms, *P* = 0.029) and C4 electrodes (from 301.11 ± 18.72 to 309.41 ± 18.13 ms, *P* = 0.023) in the sham-TNS group. This result was confirmed by the bootstrap procedure. Although the P300 amplitude tended to be higher in the prestimulation period in the sham-TNS with respect to TNS groups, none of these differences reached statistical significance. Moreover, no between-groups difference in latencies could be found. Figure 6 shows the superimposition of ERP average response to rare tones of all 19 EEG electrodes, aligned to stimulus onset, recorded before and after TNS and the corresponding average scalp potential maps obtained at the latencies of N100, P200, P300, and at 550-ms poststimulus onset.

**Figure 7:**
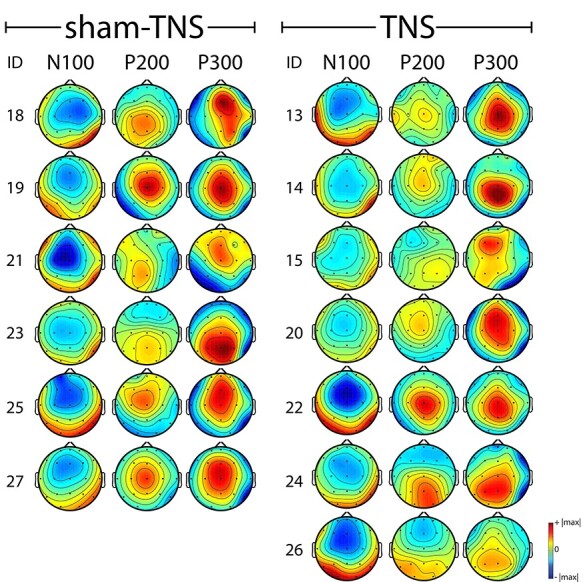
Scalp maps for each and all subjects, belonging to sham-TNS and TNS groups, showing EEG activations at N100, P200, and P300 latency. In both groups, each line corresponds to a single subject. Maps were obtained by averaging pre- and post-TNS-sham/TNS stimulation. In each subject, the range of the color scale was the same in all the scalp maps and corresponded to ±the highest absolute value observed at the 3 time points.

As shown in Figure 7, where pre- and poststimulation data were averaged, the N100, P200, and P300 waveforms were spatially consistent across subjects. The N100 and the P200 scalp maps showed greater negativity in the frontal and in the central regions, respectively. The P300 was observed diffusely from frontal to parietal regions both in the sham-TNS and in the TNS groups.

### PSD of EEG Open Eyes

**Figure 8:**
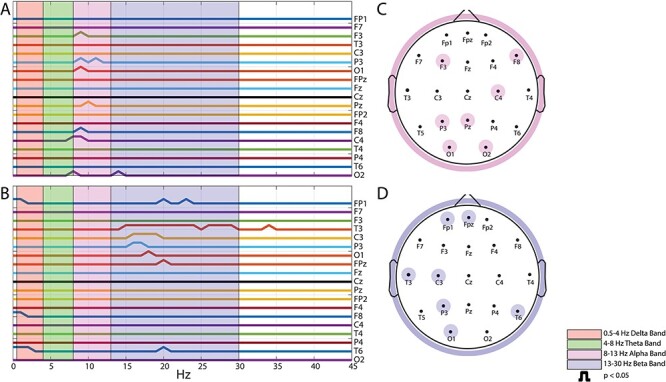
Comparison between pre- and poststimulation values of the PSD of rest EEG signal (open-eyes) relative to sham-TNS (*A*, *C*) and TNS (*B*, *D*). In *A* and *B*, each line corresponds to a single electrode. Upper deflections represent points of significant pre-/postdifferences. The highlighted areas in red, green, pink, and blue represent the delta (0.5–4 Hz), theta (4–8 Hz), alpha (8–13 Hz) and beta (13–30 Hz) band, respectively. In *C* and *D*, the highlighted electrodes show significant PSD differences in the alpha and in the beta band for the sham-TNS and TNS conditions, respectively.

Power spectra of EEG open eyes signal obtained in the pre- and poststimulation periods were compared by paired *t*-test for TNS and sham-TNS groups. As shown in Figure 8, point-by-point analysis of the power spectrum (1 Hz bins) showed that several frequency clusters displayed significant differences between pre- and poststimulation. It is worth noting that in the TNS group (Fig. 8, lower row), a significant reduction was observed at 24 frequency points within the beta band, distributed across 7 electrodes, whereas this was the case only for one point (electrode) in the same band of sham-TNS group. However, in the TNS group, the bootstrapping procedure did not confirm significance for 23 out of the 24 frequency bins.

**Figure 9:**
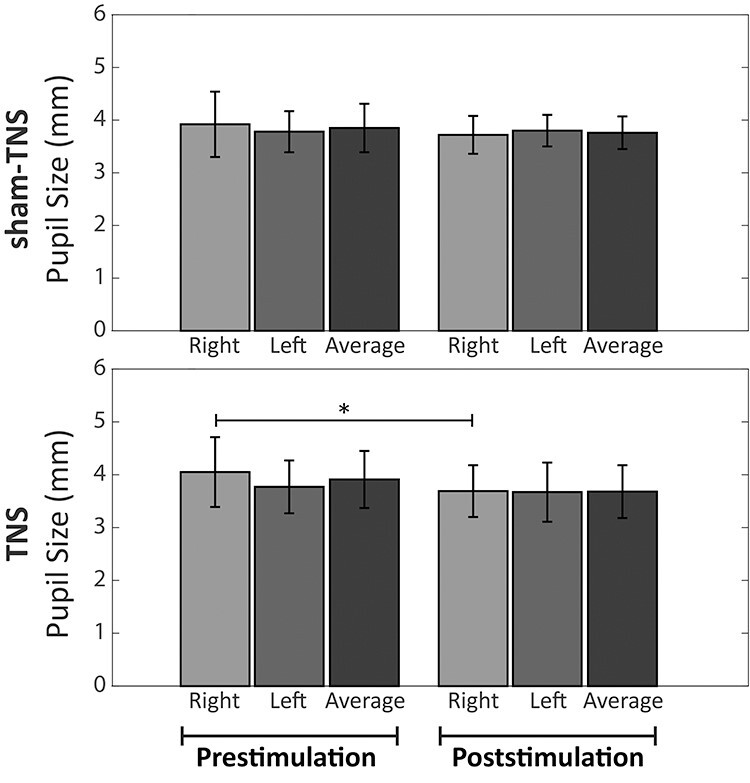
Mean ± SD of pupil size evaluated with the dental arches in contact in pre- and post-TNS/sham-TNS conditions.

**Figure 10:**
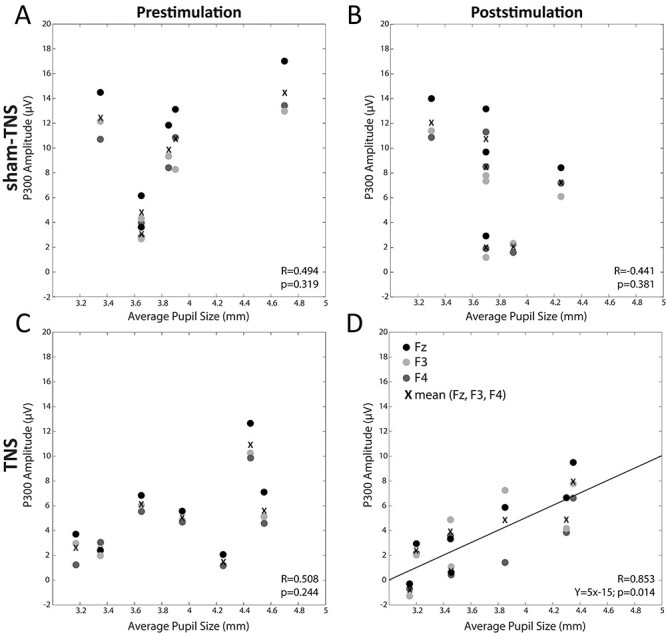
Regression analysis between the P300 amplitude in frontal electrodes and the average pupil size observed before (*A*,*C*) and after (*B*,*D*) sham-TNS (*A*,*B*) and TNS (*C*,*D*) condition. The regression lines have calculated for the average values of the 3 frontal electrodes represented in the plot, which are indicated by crosses.

On the other hand, following sham-TNS (Fig. 8, upper row), 9 frequency bins within the alpha range showed a significant enhancement. The bootstrapping procedure showed that in 6 of these bins and in 2 of the adjacent ones *P* values ranged from 0.055 to 0.025, whereas from 0.067 to 0.084 in the remaining 3.

### Pupil Size Measurements and Correlations with P300 Amplitude

Since no significant differences were observed between pupil size recorded with the dental arches apart and in contact, neither before, nor after TNS/sham-TNS, only results obtained in the contact position will be described. The average pupil size evaluated in contact position, before TNS/sham-TNS, corresponded to 3.91 ± 0.54 and 3.85 ± 0.46 mm, respectively, without significant differences between the 2 experimental groups. Details about right, left, and average pupil size values obtained with the arches in contact, pre- and post-TNS/sham-TNS are given in Figure 9.

When data obtained pre- and post-TNS/sham-TNS were compared, a significant difference emerged only for the right side of the TNS group when the teeth were in contact, pupil size being 9% smaller in the poststimulus period (Fig. 9).

The anisocoria values (absolute difference between right and left pupil size) observed in contact position before TNS and sham-TNS corresponded to 0.36 ± 0.40 and 0.37 ± 0.31, respectively. These values were reduced, following both treatments, to 0.24 ± 0.23 and 0.22 ± 0.34, respectively. These differences, however, did not reach the significance level.

The study of the correlation between pupil size and P300 amplitude was restricted to F3, C3, P3 (left side), Fz, Cz, Pz (middle), and F4, C4, P4 (right side), where the P300 was prominent (Fig. 3) (Picton 1992). So, for this analysis, the significance level acceptable for a single correlation corresponded to *P* = 0.006 (Bonferroni’s correction). This analysis was performed separately for TNS and sham-TNS groups, before and after the treatment. Before the treatment, left, average, and right pupil size values were not correlated with left, middle, and right values of P300 amplitude, neither in the TNS nor in the sham-TNS group, whatever electrode (frontal, central, and parietal) was considered.

However, following TNS, but not sham-TNS, a significant positive correlation could be found between the P300 recorded at Fz and the average pupil size (*R* = 0.905, *P* = 0.005). Individual values of P300 amplitude recorded at Fz are represented as a function of the average pupil size by black dots in Figure 10. At Fz, before TNS, the correlation coefficient was 0.546 (*P* = 0.205).

As to the sham-TNS group, no correlation was found at Fz, neither before (*R* = 0.502, *P* = 0.310), nor after the treatment (*R* = 0.448, *P* = 0.373). A further analysis was performed by averaging the P300 values of left, central, and right electrodes at frontal (F: F3, Fz, F4), central (C: C3, Cz, C4), and parietal (P: P3, Pz, P4) regions. Moreover, we also averaged the P300 values of frontal, central, and parietal electrodes located on the left (L: F3, C3, P3), on the right (R: F4, C4, P4) and on the midline (M: Fz, Cz, Pz). These averaged P300 values were correlated with the left, right, and average pupil size, respectively. In this analysis, according to Bonferroni’s correction, the significance level was *P* = 0.017. Also, for these averaged values, no significant correlation could be found in the prestimulation period both in the TNS and the sham-TNS group. In particular, in the TNS group, before the treatment, the correlation coefficients for the average frontal electrode corresponded to 0.508 (*P* = 0.245), whereas in the sham-TNS group, pretreatment value was 0.495 (*P* = 0.318).

The lack of correlation persisted following sham-TNS (*R* = −0.441, *P* = 0.381), whereas, following TNS a significant, positive correlation could be found in the frontal region (*F*: *R* = 0.853, *n* = 7, *P* = 0.015), with pupil size representing the source of 72% of P300 variability. Figure 10 shows that, although a significant correlation between pupil size and P300 amplitude was observed only for Fz, similar trends characterized also F3 and F4, well fitted by the regression line obtained for the averaged values (Fig. 10*D*).

When the averaged middle electrodes (Fz, Cz, Pz) were taken into account, in the TNS and sham-TNS group, before the treatment, the correlation coefficients corresponded to 0.547 (*P* = 0.204) and 0.408 (*P* = 0.422), respectively. Following stimulation, the correlation coefficient was unmodified in the sham-TNS group (*R* = 0.538, *P* = 0.271), whereas increased in the TNS group (*R* = 0.774, *P* = 0.041). However, in the latter instance, the corresponding *P* value was higher than the significance level imposed by Bonferroni’s correction.

### Cognitive Performance

In both TNS and sham-TNS groups, the number of errors in the oddball paradigm was not significantly different between pre- and poststimulation periods (pre-TNS: 0.33 ± 0.52; post-TNS: 0.33 ± 0.52, paired *t*-test NS; pre-sham-TNS: 0.60 ± 1.34; post-sham-TNS: 1.20 ± 1.64, paired *t*-test: not significant).

## Discussion

The present experiments show that the P300 ERPs amplitude decreases following TNS in several brain regions, whereas it was not significantly affected when subjects were resting without stimulation (sham-TNS) for a comparable amount of time. In addition, neither TNS, nor sham-TNS significantly modified the EEG average power spectrum in the delta, theta, alpha, and beta bands. However, in the sham-TNS group, localized significant enhancements in power occurred between 8 and 11 Hz, thus indicating a tendency to cortical synchronization within the alpha band during the experimental session. No major differences in pupil size were observed following both TNS and sham-TNS, except for a 9% decrease in the right pupil size following TNS. Moreover, in both the TNS and sham-TNS groups no correlation was found between P300 amplitude and pupil size before the treatment, whereas a significant, positive correlation in the frontal and midline electrode was observed following TNS but not sham-TNS.

The absence of major TNS effects on the pupil size (an indicator of basal LC activity) is consistent with the study of Tramonti Fantozzi et al. (2017), who showed that short bouts of masticatory activity do not modify pupil size at rest. This activity, however, led to long-lasting effects on cognitive performance, as TNS did on the P300 amplitude in the present experiments. However, in spite of the lack of major TNS-induced pupil size changes, TNS led to a strong correlation between P300 amplitude and pupil size in the frontal and midline brain regions, not observed before the stimulation. We may suggest that repeated trigeminal activation enhances the coupling of this ERP with the basal level of central noradrenergic modulation and that trigeminal activation, anyhow achieved (chewing or TNS), could represent a viable approach capable of modulating noradrenergic afferents, cortical excitability, and performance.

In the present experiments, the P300 amplitude was modified by TNS in frontal, central, and parietal spots. This observation suggests that trigeminal stimulation may modify neural processing in regions that play different functions in the oddball task: orienting selective attention, elaborating sensory information, storing, and retrieving information, comparing present sensory experience with memory content and, finally, mentally counting the detected rare events (Polich 2007).

Although TNS has been repeatedly tested as a noninvasive treatment for refractory epilepsy and mood disorders (DeGiorgio et al. 2003, 2006, 2013; Aaronson et al. 2013), only another study—to the best of our knowledge—has so far investigated its effect on the P300 amplitude/latency in healthy subjects (Gadeyne et al. 2016). In this paper, the ophthalmic trigeminal branch was activated without significant effects on the P300 amplitude. So, the effects observed in the present experiments could be attributed to the activation of inputs from muscle spindles and periodontal receptors traveling within the mandibular branch.

The extensive connections of the trigeminal input with LC neurons (De Cicco et al. 2018) and the dependence of the P300 generation upon the central noradrenergic system (Nieuwenhuis et al. 2005) are the anatomofunctional ground possibly explaining the present results. However, the knowledge about trigeminal–LC relationships would have suggested an enhancement, rather than a decrease in P300 amplitude following TNS. It is known, in fact that, in subjects showing an asymmetric masseter EMG activity during clenching, pupil size—a fine indicator of the level of LC activity—is larger on the side of higher trigeminal sensorimotor activity (De Cicco et al. 2018), suggesting an excitatory trigeminal input to the ipsilateral LC. Moreover, the P300 is related to phasic LC activity, leading to noradrenaline release from axon terminals (Nieuwenhuis et al. 2005), as indicated by its sensitivity to noradrenergic drugs (Swick et al. 1994; Brown et al. 2015; de Rover et al. 2015) and by the P300 amplitude decrease after LC lesion in monkeys (Pineda et al. 1989). Based on these data, suggesting that an higher noradrenergic activity corresponds to a larger P300 amplitude, it was reasonable to expect an increased P300 amplitude following TNS.

Several factors may modify the P300 amplitude, such as the frequency of the targets (Croft et al. 2003), the probability of the rare events in the oddball sequence (Nieuwenhuis et al. 2005) and the discriminability between rare and frequent events (Picton 1992): all these parameters, however, were kept constant across the experimental sessions. In particular, the ratio between target/standard stimuli always corresponded to 1/4 (Polich 2007).

Moreover, the P300 amplitude is usually increased by motivational instructions that enhance the attention to the task (Carrillo-de-la-Peña and Cadaveira 2000) or by adding another cognitive task to distinguish rare and frequent tones (Alexander et al. 2005). However, none of these confounding factors were present in our experiments.

Finally, the P300 amplitude depends upon the level of arousal and basal LC activity, as indicated by simultaneous pupil size and ERPs recordings during oddball tasks (Murphy et al. 2011) but, in the present study, the decrease in the P300 amplitude was not associated to major changes in pupil size.

Given the relation between LC activity, P300 amplitude and cognitive performance, the results of this study could be explained when taking into account that, in our subjects, the performance was at the ceiling (>99% of correct reports of rare stimuli) and was not modified, neither by TNS, nor by sham-TNS.

On the bases of our findings, we can speculate that a preceding period of TNS leads to a large release of noradrenaline at cortical level during target discrimination, which modifies the processing of cortical networks and promotes an improvement in detection/recognition skills (Gelbard-Sagiv et al. 2018; Devilbiss 2019). This enhanced efficiency of cortical circuits may persist also when LC activity is back to the prestimulation levels (Klukowski and Harley 1994; Tramonti Fantozzi et al. 2017) and could be related to the signal-to-noise ratio enhancement (Foote et al. 1975; Moxon et al. 2007; Devilbiss 2019) in brain regions involved in perceptual processing (Fazlali et al. 2016). As a result, once the trigeminal nerve has been activated, a lower network activity is necessary for obtaining the same discriminative outcome. In this respect, noradrenaline often reduces both spontaneous and evoked activity when applied to individual neurons (Kasamatsu and Heggelund 1982; Kolta and Reader 1989; Manunta and Edeline 1997; Ego-Stengel et al. 2002; Ikeda et al. 2015). This would result in a lower cortical activation during target recognition, and, in particular, in a lower amplitude of P300, specifically related to this process. This hypothesis is consistent with the assumption that the P300 amplitude reflects the amount of useful sensory information transmitted during perception (Picton 1992). A lower activation in cortical motor areas also underlines skill acquisition in sensorimotor processes (Saling and Phillips 2007; Wu et al. 2008). In this respect, a functional magnetic resonance imaging study (Tramonti Fantozzi et al. 2019) has shown that finger movement executed with a balanced trigeminal input were associated with lower neural activation, which is indicative of higher skill and less attentive effort during motor performance.

Since we did not perform any recording during TNS, we cannot exclude an initial increase in P300 amplitude, due to the enhanced norepinephrine release elicited by the stimulation. However, it is reasonable to assume that the drop in P300 amplitude, observed after TNS, begins during the stimulation and persists after its end.

The results can be also interpreted within the framework of the “adaptive gain theory” proposed by Aston-Jones and Cohen (2005), stating that phasic LC activation during task enhances cortical processing of task-related stimuli, boosting performance, whereas a very low or high basal level of tonic LC activity decreases LC phasic activation promoting task disengagement. It is likely that TNS enhances LC phasic activation, in a manner similar to that achieved by 2 min of chewing (Tramonti Fantozzi et al. 2017). Moreover, the tonic LC activity level does not seem to be modified after chewing and only slightly after TNS. However, following TNS, the underlying enhancement in LC phasic activation could not lead to a performance improvement, already at ceiling. However, the enhanced phasic release of norepinephrine might increase the efficiency of cortical processing leading to a lower neural engagement for achieving an identical task performance.

It still has to be established whether TNS effectively enhances phasic pupil dilation following rare stimuli and whether a reduction of P300 by TNS also underlies more difficult tasks, where the discrimination between target and frequent stimuli has been made challenging by reducing the corresponding difference in spectral content, so that the performance is not at ceiling. Additional studies, applying more demanding cognitive tasks, are necessary to further support the promising perspective of a clinical application. The preliminary observations described in the present report, however, suggest a possible use of the TNS for improving cognitive performance in patients affected by cognitive disorders or ARAS dysfunctions.

## Notes

We would like to thank Mrs Cristina Pucci, Mr Francesco Montanari, and Mr Paolo Orsini for their technical support. We are grateful to Dr Silvano Presciuttini for a useful discussion about the statistical approach, to Dr Federico Cucchiara, and to Dr Tommaso Banfi for helping setting up data.

*Conflict of Interest*: None declared.

## Funding

ARPA Foundation, Pisa, Italy (liberal donation to U.F.), the Italian Ministry of Health (grant GR-2011-02348998 to U.F.); University of Pisa, Pisa, Italy (Fondi di Ateneo to D.M., P.d.A, L.B., U.F.).
